# Supplementary figures and images for: PDGFR-β signaling mediates HMGB1 release in mechanically stressed vascular smooth muscle cells
Source: PLoS One. 2022 Mar 16;17(3):e0265191. doi: 10.1371/journal.pone.0265191 (PMC8926240; doi:10.1371/journal.pone.0265191)

<Nucleus>

MS, 3%

0 0.5 1 hr

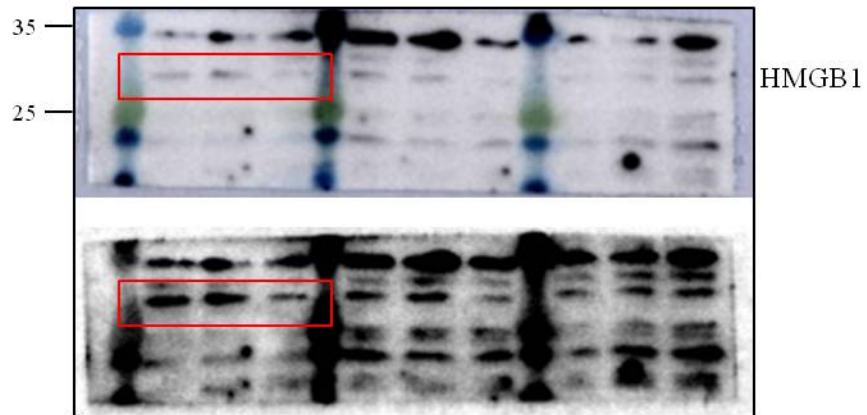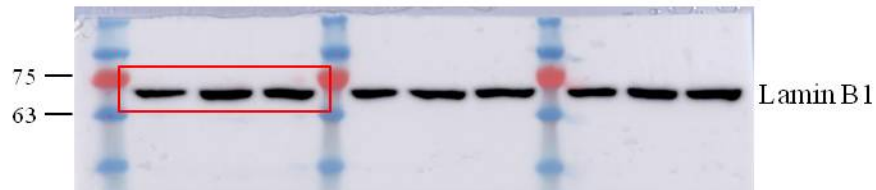

<Cytosol>

MS, 3%

0 0.5 1 hr

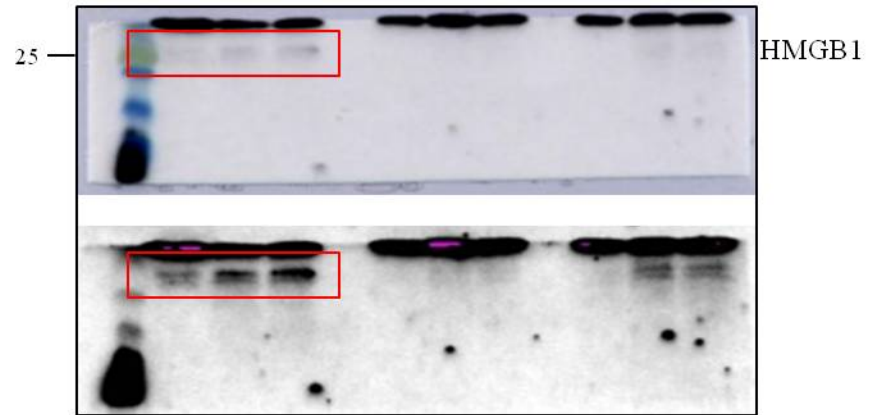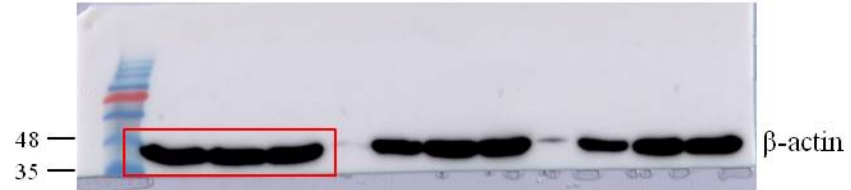

Supplement: S1 Raw images — (PDF) [file pone.0265191.s001.pdf]

IP (HMGB1)

Input (30  $\mu$ g)

Static  
3% MS, 0.5 hr  
3% MS, 1 hr

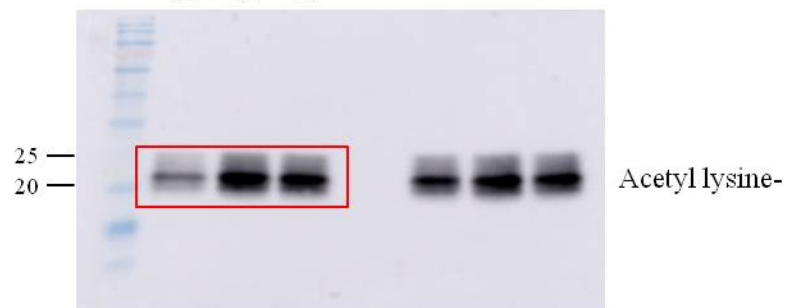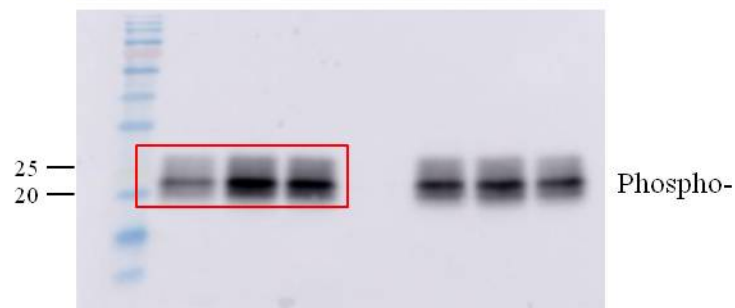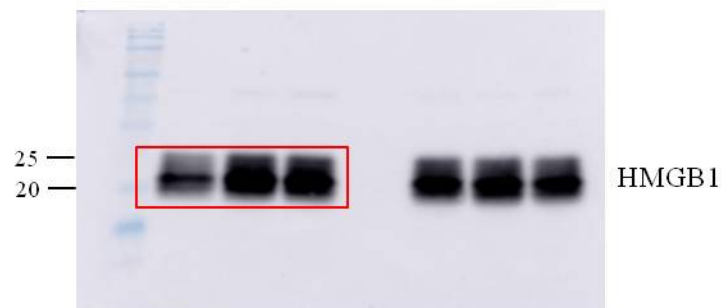

Static  
3% MS, 0.5 hr  
3% MS, 1 hr

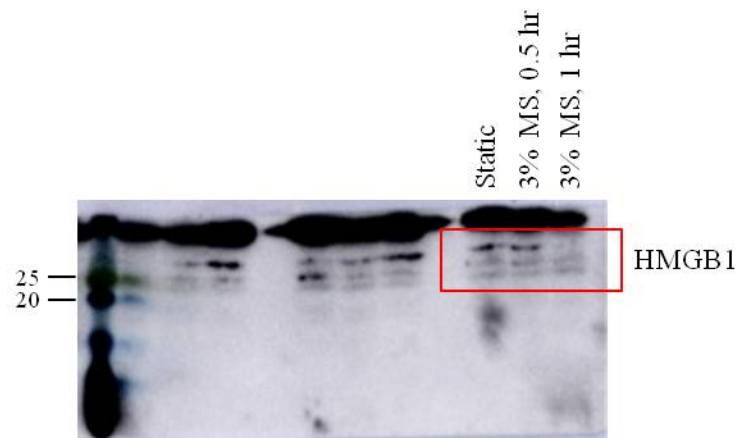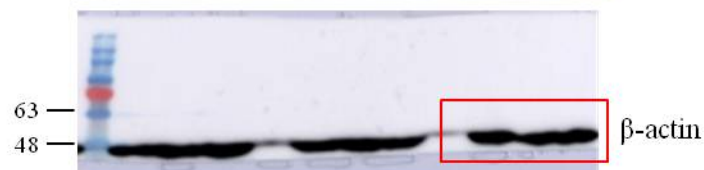

Supplement: S2 Raw images — (PDF) [file pone.0265191.s002.pdf]

MS, 3%  
0 1 hr

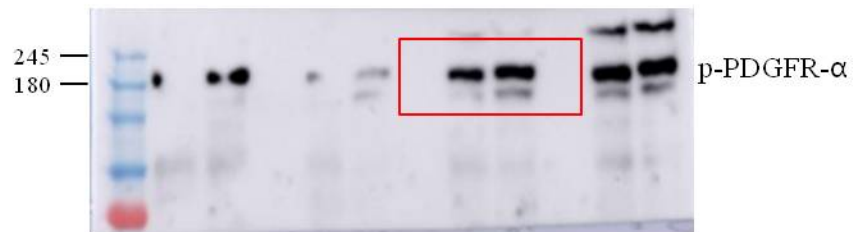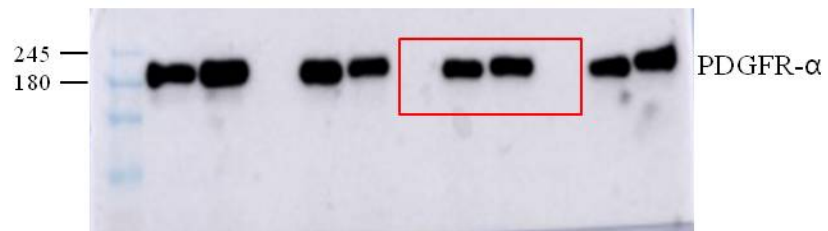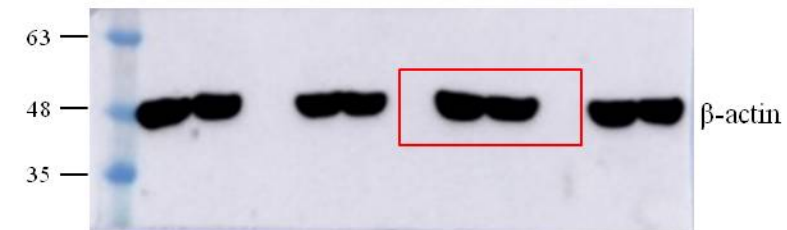

MS, 3%  
0 1 3 6 9 12 hr

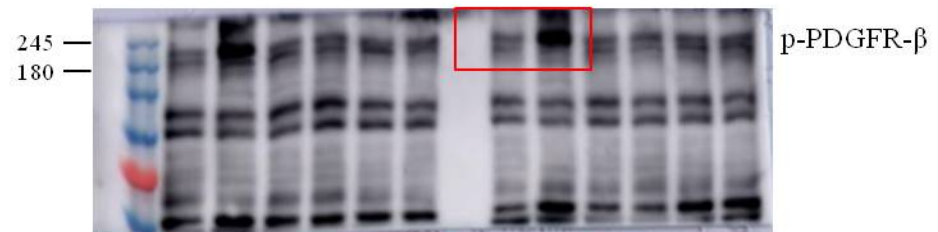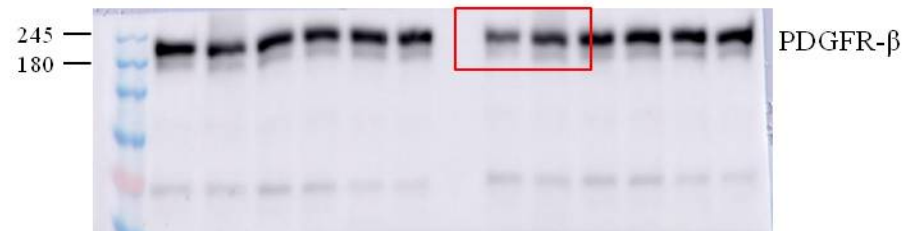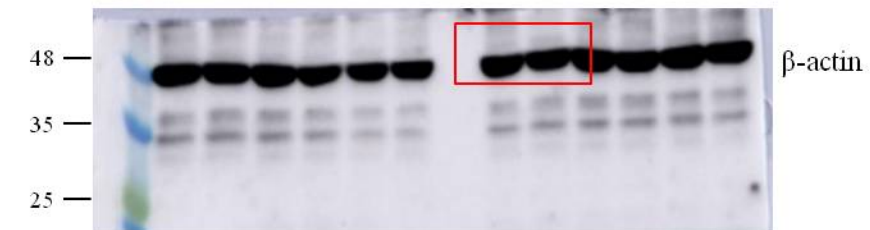

Supplement: S3 Raw images — (PDF) [file pone.0265191.s003.pdf]

1\_ Negative Control  
 2\_ PDGFR- $\alpha$  siRNA  
 3\_ Negative Control  
 4\_ PDGFR- $\alpha$  siRNA

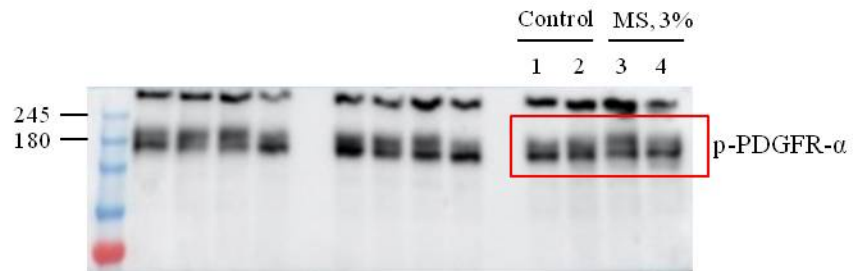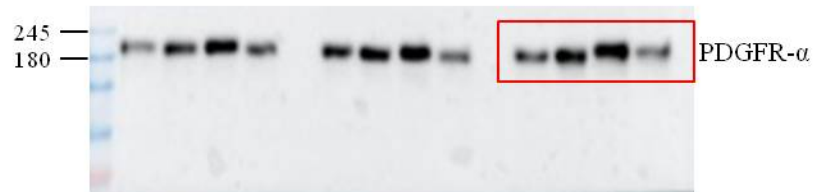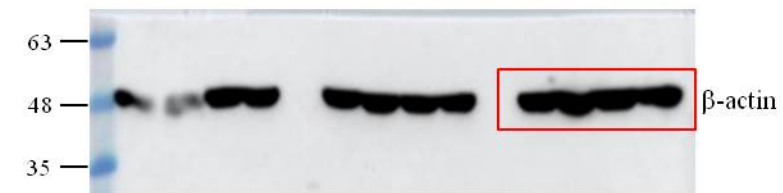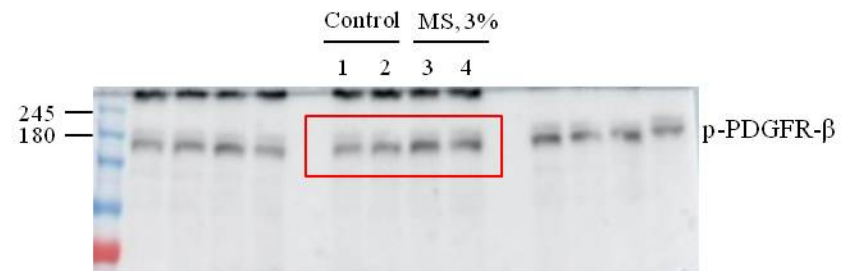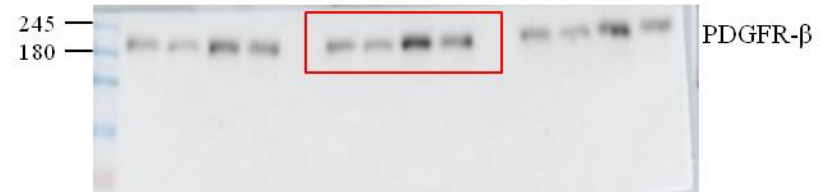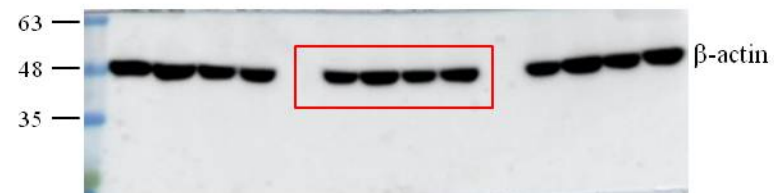

Supplement: S4 Raw images — (PDF) [file pone.0265191.s004.pdf]

PDGF-AA, ng/ml

0 1 5

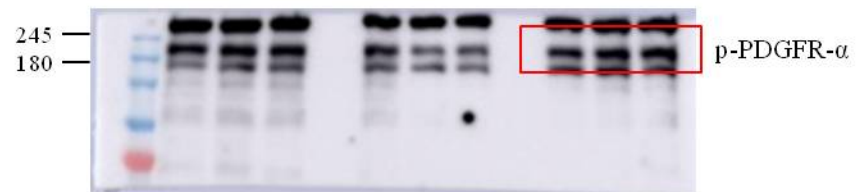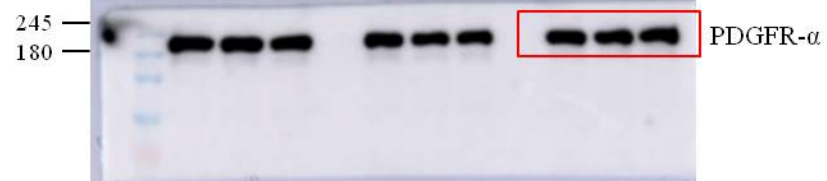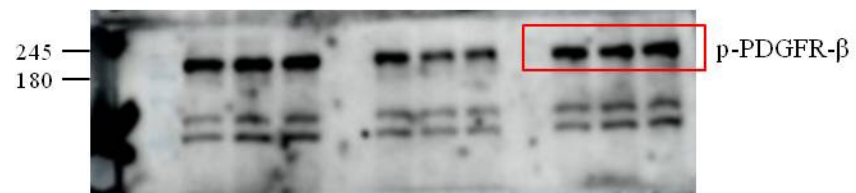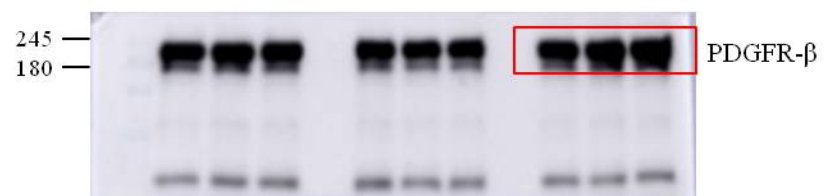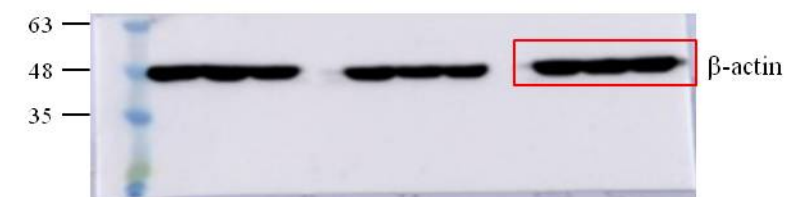

PDGF-DD, ng/ml

0 1 5

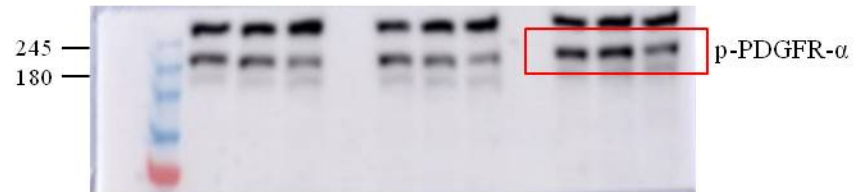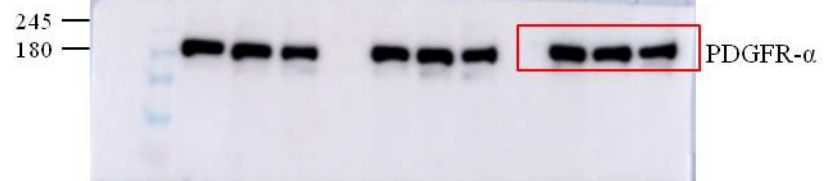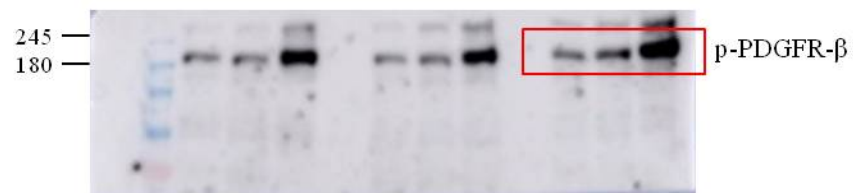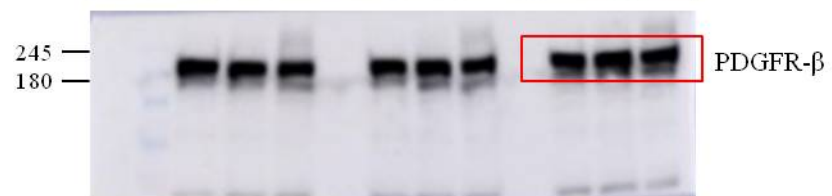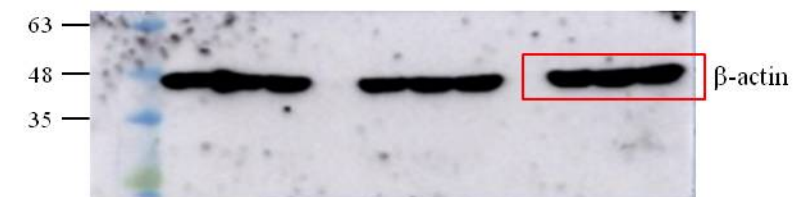

Supplement: S5 Raw images — (PDF) [file pone.0265191.s005.pdf]
